# Systemic treatment with resveratrol reduces the progression of experimental periodontitis and arthritis in rats

**DOI:** 10.1371/journal.pone.0204414

**Published:** 2018-10-03

**Authors:** Mônica G. Corrêa, Paula Rodrigues Pires, Fernanda Vieira Ribeiro, Suzana Peres Pimentel, Fabiano Ribeiro Cirano, Marcelo Henrique Napimoga, Marcio Z. Casati, Renato Corrêa Viana Casarin

**Affiliations:** 1 Dental Research Division, School of Dentistry, Paulista University, São Paulo, São Paulo, Brazil; 2 Laboratory of Immunology and Molecular Biology, São Leopoldo Mandic Institute and Research Center, Campinas, SP, Brazil; New York Medical College, UNITED STATES

## Abstract

Rheumatoid arthritis and periodontitis are chronic inflammatory diseases which has been closely associated due to the nature of immune-inflammatory imbalance response. Resveratrol is a naturall product with biological proprieties that may promote immunomodulatory effects on host response. This study investigated resveratrol continuous administration effect on experimental periodontitis and arthritis progression in rats. Thirty-five rats were assigned to the following groups: 1—experimental arthritis + experimental periodontitis + placebo (RA+EP +PL) (n = 12); 2 –RA+EP+ ibuprofen (RA+PE+IB) (n = 11); 3—RA+EP+ resveratrol (RA+PE+RSV) (n = 11). After euthanasia, the specimens were processed for morphometric analysis of bone loss, and the gingival tissue surrounding the first molar was collected for quantification of inflammatory markers using a Luminex/MAGpix assay and anti-citrullinated protein antibody (ACCPA) levels were measured by ELISA assay. Serum level of rheumatoid factor (RF) was measured by ELISA assay. Paw edema was analyzed using a plethysmometer. Higher bone loss was observed in PL group, when compared to IB and RSV groups. RSV group presented higher IL-4 concentration than PL and IB groups. Resveratrol reduced RF serum levels and both IB and RSV decreased ACCPA gingival levels. Besides, paw swelling level was significantly lower in IB and RSV groups in the 21^th^ day and only in RSV group in the 28^th^ day. Histological analyzes showed smooth articular surface and higher width of the subchondral cortical in RSV group. Resveratrol showed modulatory effect and seems to reduce the inflammatory signs of arthritis and articular damage throughout the time.

## Introduction

Rheumatoid arthritis is a chronic inflammatory autoimmune disease with a prevalence of 0.5% to 1.0% in adults in industrialized countries that results in bone and cartilage loss [1]. On the other hand, severe periodontitis has a prevalence of 10% a 15% [2] which can result in tooth loss. Both conditions are host-mediated by production of exacerbated immune-inflammatory mediators, which lead to tissue destruction and to the maintenance of the inflammatory profile [3, 4]. Periodontal disease is a bacterial and chronic inflammatory condition that leads to the occurrence of supporting tissue destruction [3]. Moreover, different studies have associated the oral bacterial infection (bacterial load or serum titre measurement) and RA trigger and progression [5].

Relevant clinical studies, with considerable number of included subjects, have shown worst periodontal condition, higher risk of edentulism, higher prevalence of moderate and severe periodontitis, bone loss, percentage of deep pockets, greater attachment loss in RA patients compared to systemic health patients [6–11]. A recent systematic review evaluated the association between RA and periodontitis including 17 papers with a total of 153,492 patients. The authors observed increased risk of periodontitis in the presence of RA compared to healthy controls (relative risk: 1.13; 95% CI: 1.04, 1.23) with significantly worst clinical periodontal parameters in those with RA [12]. These findings are also confirmed in several animal studies, which show more bone loss and higher levels of inflammatory cytokines in the presence of RA [13–16].

As previously discussed [13], T lymphocytes, macrophages and polymorphonuclear infiltration occur in RA and in periodontitis causing progressive tissue destruction [3, 4, 17]. The maintenance of the inflammatory process is mediated by cytokines (TNF-α, IL-1β, IL-6, IL-8, IL-12, IL-17, IL-18, IL-23 and IFN-γ) in RA and in periodontitis [3, 17–19]. The bidirectional relationship between both diseases has been investigated and studies have showed increase in gingival inflammatory status in the presence of RA [9–11], as well as the increasing of RA occurrence and severity in a periodontitis condition [20]. Considering this, immunomodulation has been a therapeutic approach evaluated [21] for both diseases, especially focusing on natural products [22–28].

Resveratrol (3, 4’,5-trihydroxystilbene), a pleiotropic molecule, is a polyphenol not flavonoid, antifungal plant-derived substance that also is present in food like grapes, cranberries and peanuts [29]. Resveratrol has attracted great attention due to several biological properties as improvement of metabolic control of diabetes [30], anti-cancer activity [31], antioxidant enzyme activities [30, 32], protection against neural degeneration [33], and prevention of cardiovascular diseases [34]. Additionally, resveratrol may positively interfere with osteoblastogenesis, contributing to new bone formation [35]. Considering its effect on periodontitis progression, animal studies have showed reduced alveolar bone loos and imuno-modulatory effect [22, 36]. Besides, a protective role of resveratrol on collagen-induced arthritis in mice related to its inhibitory effect on Th17 cell expansion and IL-17 production was observed. Studies in animals [37–39] and in humans [40–42] demonstrated an effective property of resveratrol in suppressing inflammatory activities. However, to the author’s knowledge, there is no study investigating the effect of resveratrol on the progression of both RA and periodontitis associated.

In face of the above evidence, this study investigated the effect of continuous use of resveratrol systemically administered on the progression of experimental periodontitis and arthritis, on the local and serum cytokine levels and on the clinical and articular feature of RA condition.

## Material and methods

### Animals

The animals were composed of 34 adults male Wistar rats, weighing between 200 to 300g (Butantan Institute in São Paulo, Brazil) at the beginning of the study. There was a 15 days period of acclimatization before use and the animals were kept in temperature-controlled cages, with access to food and water ad libitum. The experimental procedure was approved by the Paulista University Institutional Animal Care and Use Committee (205/13 CEP/ICS/UNIP).

### Experimental design

#### Treatment groups

**Fig 1:**
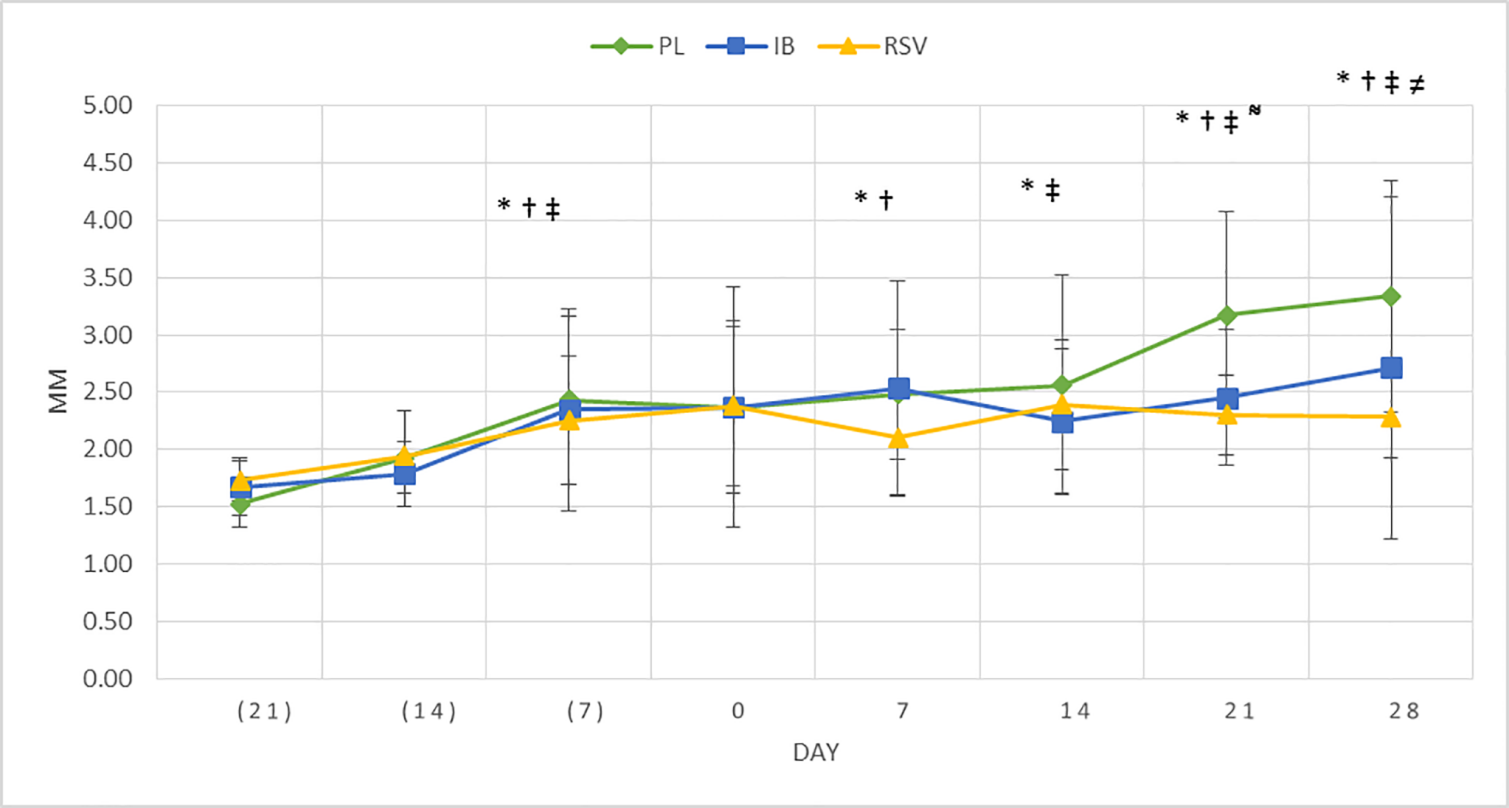
Means a± SD of paw swelling (volume—ml) measured by plethysmometer.

The experimental design can be observed in Fig 1. The animals were assigned to one of the three groups: 1- experimental arthritis + experimental periodontitis + placebo (RA+ EP + PL) (N = 12); 2—experimental arthritis + experimental periodontitis + ibuprofen (RA+ EP+IB) (N = 11); 3—experimental arthritis + experimental periodontitis + resveratrol (RA+EP+RSV) (N = 11). Treatments consisted in daily administration of a placebo solution, 30 mg/kg of ibuprofen [43, 44] and 10 mg/kg of resveratrol [13, 22, 36]. A stock solution of resveratrol (R5010-500MG; Sigma-Aldrich, São Paulo, São Paulo, Brazil) (molecular weight = 228.2) was prepared in 100 mL of Tween-80 (P4780; Sigma-Aldrich, São Paulo, São Paulo, Brazil) and further dilutions of both were made in water to obtain the concentrations required for this investigation. A stock solution of ibuprofen (Brainfarma Indústria Química e Farmacêutica S.A, Anápolis, Goiás, Brasil) was prepared in 1000 mL of water. The placebo solution was composed of the same quantities of Tween-80 and water, as used in the preparation of resveratrol. The therapies were administered via gavage for 30 days–from day 0 to day 30. The animals were evaluated daily throughout the experiment to check for possible clinical or toxicological symptoms. This analysis was performed by weighting the animals 3 times/week, checking daily the food and water consumption and examining clinically any sign of illness different from those caused by AR.

#### Rat arthritis model

As described in detail previously [13], RA was induced by two immunizations of a type-II collagen (CII) (CII; C9879-1G - Sigma-Aldrich, São Paulo, São Paulo, Brazil) emulsion in Incomplete Freund’s Adjuvant (IFA—F5506–10 mL; Sigma-Aldrich, São Paulo, São Paulo, Brazil) and a third immunization with Complete Freund’s adjuvant (CFA—F5881–10 mL; Sigma-Aldrich, São Paulo, São Paulo, Brazil). The fist injection was composed by a total of 300 μg of CII in IFA in several points of the tail base and the second dose (booster dose) consisted of 100 μg of CII in IFA, subcutaneously. The third immunization included two injections of CFA (1mg/ml) made in the paw subcutaneously (0.1 ml) and in the knee joint via intra-articular (0.1 mL).

#### Rat periodontitis model

Experimental periodontitis was induced in one of the mandibular first molars of each animal through the placement of cotton ligature (Corrente Algodão 10, Coats Corrente, São Paulo, São Paulo, Brazil) knotted subgingivally at the cementoenamel junction, as previously described [13,22]. The presence of the ligatures submarginally permitted the biofilm accumulation over 30 days. The contralateral teeth did not receive ligatures and were used as a control. The animals were anesthetized by the intramuscular administration of ketamine hydrochloride (0.5 mL/kg) and xylazine hydrochloride (10 mg/kg).

#### Euthanasia and specimens collecting

The euthanasia occurred 51 days after the beginning of the study using CO_2_ inhalation and the mandibles were excised for morphometric analysis. The buccal gingival tissue from the area surrounding the lower first molar subjected to experimental periodontitis was also collected for immune-inflammatory analysis using Luminex/MAGpix assay.

#### Measurement of alveolar bone loss

The specimens were prepared after gingival dissection, as described in detail previously [13, 22]. Briefly, the mandibles were de-fleshed after immersion in 8% sodium hypochlorite for 4 h and then the specimens were washed in running water and dried with compressed air. An 1% aqueous methylene blue solution (Sigma-Aldrich, St. Louis, MO) was applied to the specimens to distinguish the cementum enamel junction (CEJ) and after this they were washed in running water. Photographs of the buccal aspects were taken, and representative linear alveolar bone loss was assessed on the buccal surface of the lower first molars by measuring the distance of the CEJ from the alveolar bone crest at three equally distant sites. To validate measurement conversions, all specimens were photographed alongside a millimeter ruler [13, 22, 23, 36]. A single examiner (PRP), who was blinded to the experimental group identities, carried out morphometric measurements. The measurements were performed after intraexaminer calibration by evaluating 10 images twice within 24 hours, obtaining an intraclass correlation of 91%.

#### Immunoenzymatic assay

This procedure was carried out as described previously by the authors of the present study [13]. The collected tissues were placed into sterile tubes containing 400μl phosphatebuffered saline (PBS) with 0.05% Tween-20 and the samples were stored at -20°C until the analysis. Next, the tissue was weighed, cut into small pieces (1 mm^3^ to 2 mm^3^), and solubilized in PBS (100 mg tissue/ml). The levels of IL-4, IL-1β, IL-6, IL-17 and TNF-α were determined by Luminex/MAGpix assay using commercially available kits (RCYTOMAG-80K; Millipore, Billerica, MA, USA) and following the manufacturers’ instructions. The standard curve range used for IL-1β measurement was 2.4–10,000 pg/ml; for IL-4 measurement, 4.9–20,000 pg/ml; for IL-6 7.3–300,000 pg/ml, for TNF-α 2.4 to 10,000 pg/ml; and for IL-17 7.3 to 30,000 pg/ml.

#### Rheumatoid Arthritis factor serum levels and anti-citrullinated protein antibody ginvival levels

RF serum levels and ACCPA gingival levels were performed as described before [13]. Blood was collected from eye arteria at day 0 (T1) and day 30 (Euthanasia–T2) and the serum samples were stored at −70°C. For some animals, it was not possible to collect the necessary blood volume for the analysis. The gingival tissue was prepared as described above for multiplex immunoenzymatically assay and some samples were lost during the assay conduction. The level of RF and anti-citrullinated protein antibody in serum aliquots and gingival tissue were measured by using Rat Elisa Kits (MBS720877—monoclonal anti-RF antibody and an RF-HRP conjugate; Mybiosorce, San Diego, California, USA; E-EL-R1431—monoclonal antibody specific to and Avidin-Horseradish Peroxidase (HRP) conjugate -Elabscience, Beijing, China, respectively).

#### Paw swelling analysis

For the assessment of paw swelling, the measurement was performed weekly using a volume displacement plethysmometer (Ugo Basile, Varese, Italia) [13].

#### Histological procedure

The whole knee joints, including synovium, adjacent tissues and bones were collected and preserved in 10% formalin solution. The joints were transversely sectioned (4–5 mm thick sections) and stained with standard Hematoxylin and Eosin (H&E). Sections were viewed by the aid of a light microscope attached with a digital camera. It was performed a descriptive evaluation concerning the joint cartilage (intact, smooth or irregular articular surface), the quantity of congested vessels and the width of subchondral cortical.

### Statistical analysis

Kruskal-Wallis/Dunn (intergroup) and Paired t test were performed (intragroup) for alveolar bone loss and cytokine levels. RF serum levels and gingival ACCPA levels were analyzed by Wilcoxon (intragroup) and Kruskal-Wallis/ Dunn tests (intergroup). In addition, a two-way ANOVA/Tukey test was used for plethysmometer values. The significance level for all analyses was 5% (Statistical Analysis System–SAS—9.3, Cary, NC, USA).

## Result

### Clinical and histological analysis

The only sing of systemic illness observed during the experimental period consisted of RA and loss of weight or deaths were not observed. In accordance with our previous study using this RA model [13], 21 days after immunization, joint swelling was observed first in the hind paws, and then joint swelling extended to the forelegs and tail. The peak of the disease occurred on day 28, with multiple and symmetrical joint swelling and redness, and the greater paw volume was noted at the paw that received the third immunization. Deformity and limited mobility were observed in some joints of some animals.

Regarding the plethysmometer analysis, increase in swelling after RA induction was observed confirming the efficacy of the RA model through the significant difference among the values of baseline (-21) and days: -7, 7, 14, 21 and 28 for placebo group; days -7, 7, 21 and 28 for ibuprofen group; days -7, 14, 21 and 28 for RSV group (p<0.05). At day 21, ibuprofen and RSV groups showed lower levels of paw edema than the placebo group (p<0.05) and at day 28 only RSV group showed a reduction in paw swelling (p<0.05) (Fig 1). However, RA symptoms (joints swelling and deformities) were observed throughout the experimental period in all groups, being the RSV group with less pronounced symptoms. Additionally, signs of gingival inflammation (redness, swelling, bleeding) were confirmed clinically during the euthanasia around the ligated teeth of all groups, with no signs of inflammation at the non-ligated sites.

**Fig 2:**
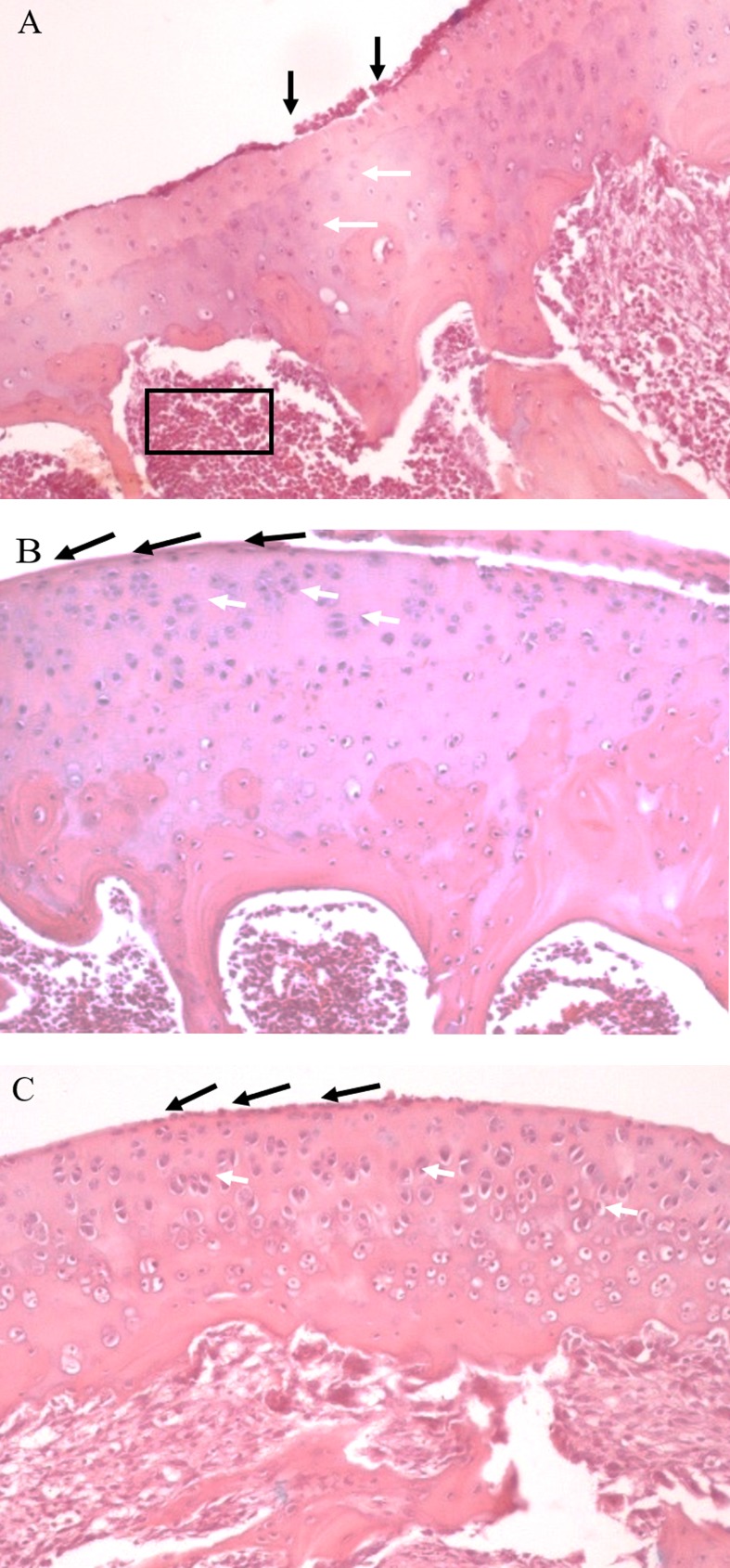
Representative photomicrographs of articular cartilages of the different groups.

Histologic evaluation of the joint in placebo group showed irregular articular surface, wider articular cartilage and chondrocytes showing pyknotic nuclei. It was also observed congested vessels. Ibuprofen group showed a smooth articular surface with thickened articular cartilage, but not as thick as in the placebo group. Besides, the chondrocytes show hyper cellularity. RSV group presented smooth articular surface. The articular cartilage shows hyper cellularity and aggregation of chondrocytes. Fig 2 illustrates the histological aspect of the joints.

### Morphometrical results

**Fig 3:**
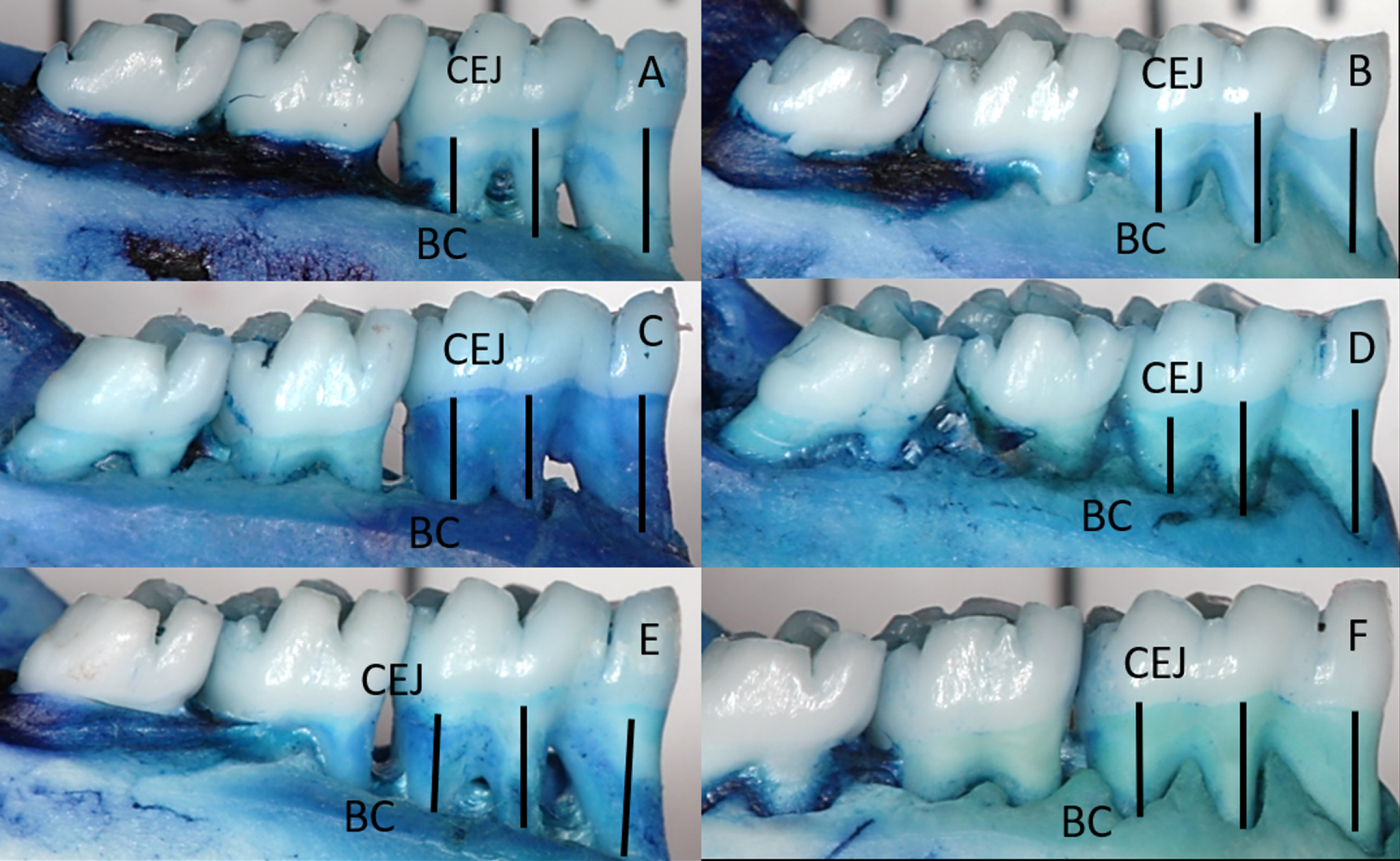
Representative photographs illustrating the morphometric findings of the groups.

Intergroup comparison of the morphometric outcomes revealed higher bone loss values in RA+EP+PL (p < 0.05) when compared to RA+EP+IB and RA+EP+RSV. There was no difference in bone loss values between RA+EP+IB and RA+EP+RSV groups (p>0.05). There was no difference between groups in unligated teeth (p>0.05). The morphometric findings are shown in Table 1 and Fig 3 illustrates the morphometric findings.

**Table 1:** Mean ± SD of alveolar bone loss (millimeters) for ligated and unligated teeth.

	MEAN LIGATED±SD	MEAN UNLIGATED±SD	*p-value* *(intragroup)*
RA+EP+PL (N = 12)	1.61±0.07 *†	1.24±0.09	*0*.*0001*
RA+EP+IB (N = 11)	1.52±0.06 *	1.27±0.07	*0*.*0001*
RA+EP+RSV (N = 11)	1.48±0.09 *	1.21±0.12	*0*.*0001*
***p-value (inter-group)***	*0*.*0014*	*0*.*3230*	

*Significant difference for intragroup comparison by Paired T test—p < 0.05.

†Significant difference for RA+EP+IB and RA+EP+RSV when compared to RA+EP+PL by (ANOVA/Tukey test; p<0.05).

No significant intergroup differences were observed from ANOVA, p > 0.05 to unligated sites.

### Gingival tissue interleukin and ACCPA levels

Analysis of inflammatory markers in gingival tissues collected at the end of the experimental period (T2) showed higher levels of IL-4 in the RA+EP+RSV group than in the control and ibuprofen groups (p<0.05). Placebo group had lower concentration of IL-4 in the ligated sites than in the unligated sites (p<0.05). Intergroup analyzes of IL-1β, IL-6, IL-17 and TNF-α revealed no differences among the groups (p>0.05) (Table 2). Besides, the RA+EP+PL group had the highest values of ACCPA (p<0.05), and ibuprofen promoted a greater reduction than RSV in these levels (p<0.05). Values are demonstrated at Table 3.

**Table 2:** Means ± SD of IL-4, IL-1β, IL-6, IL-17 and TNF-α concentrations (picograms per milliliter) measured by multiplex assay.

	IL—4		IL-1β		IL-6		IL-17		TNF-α	
*Group*	*Ligated*	*Unligated*	*Ligated*	*Unligated*	*Ligated*	*Unligated*	*Ligated*	*Unligated*	*Ligated*	*Unligated*
RA+EP+PL (N = 11)	0.03±0.03*	0.07±0.05	0.66±0.55	0.49±0.42	0.30±0.46	0.17±0.14	0.10±0.14	0.08±0.04	0.03±0.03	0.04±0.02
RA+EP+IB (N = 11)	0.08±0.08	0.10±0.12	1.52±0.96*	0.68±0.61	0.12±0.10	0.41±0.38	0.21±0.24	0.14±0.12	0.05±0.06	0.04±0.04
RA+EP+RSV (N = 9)	0.10±0.10 †	0.10±0.08	0.95±0.69*	0.41±0.34	0.27±0.37	0.20±0.23	0.13±0.02	0.09±0.04	0.04±0.02	0.04±0.01
***p-value***	*0*.*257*	*0*.*661*	*0*.*064*	*0*.*528*	*0*.*856*	*0*.*291*	*0*.*327*	*0*.*736*	*0*.*296*	*0*.*501*

*Significant difference for intragroup comparison by Paired T test—p < 0.05.

†Significant difference for RA+EP+RSV when compared to RA+EP+PL by Kruskal-Wallis/Dunn test–p <0.05.

No significant inter-group differences were observed from the Kruskal-Wallis test, p > 0.05 to unligated sites.

**Table 3:** Means ± SD of anti-citrullinated protein anti-body (ACCPA) gingival levels (U/ml) measured by ELISA assay.

*GINGIVAL TISSUE*	
*Group*	
RA+EP+PL (N = 5)	50.96±13.60 †
RA+EP+IB (N = 5)	0.02±0.01
RA+EP+RSV (N = 5)	14.28±3.32 ‡
***p-value***	*< 0*.*0001*

† Significant difference for RA+EP+IB and RA+EP+RSV when compared to RA+EP+PL (ANOVA/Tukey test; p<0.05).

‡ Significant difference for RA+EP+IB when compared to RA+EP+RSV (ANOVA/Tukey test; p<0.05).

### RF serum levels

The levels of RF in T1, prior to EP, presented no difference among the groups (p<0.05). The levels of RF increased from T1 to T2 (time of euthanasia) in RA+EP+PL and in RA+EP+IB (p<0.05), but not in RA+EP+RSV (p>0.05). Additionally, at T2 the RSV group presented lower concentration of RF than placebo and ibuprofen groups (p<0.05). (Table 4).

**Table 4:** Means ± SD of rheumatoid factor (RF) serum levels (U/ml) measured by ELISA assay.

	*T1*	*T2*	*p-value (intragroup)*
RA+EP+PL (N = 8)	71.88±4.48 *	78.06±3.07†	*0*.*26*
RA+EP+IB (N = 8)	67.97±3.30 *	76.49±3.31	*< 0*.*0001*
RA+EP+RSV (N = 8)	68.25±7.81	72.66±4.23	*0*.*166*
***p-value (inter-group)***	*0*.*187*	*< 0*.*05*	

*Significant difference for intragroup comparison (Paired T test—p < 0.05).

†Significant difference for RA+EP+RSV when compared to RA+EP+PL in T2 (ANOVA/Tukey test; p<0.05).

## Discussion

The link between rheumatoid arthritis and periodontitis and the bidirectional pathway of their development has been reported by important clinical studies [6–11, 45–47] and systematic reviews [12, 48–50]. One disease seems to alter the imuno-inflammatory response of another, such as the concentration of pro-inflammatory cytokines, RF and ACCPA serum and gingival levels [13]. The effect of resveratrol has been investigated in the treatment of both RA [51–52] and periodontitis [22, 36], but not when the diseases are associated. This study evaluated the influence of systemic treatment with resveratrol on the progression of both diseases. Resveratrol and ibuprofen promoted lower alveolar bone loss and lower gingival levels of ACCPA than placebo. Only resveratrol was capable to modulate the local levels of IL-4 and the serum levels of rheumatoid factor. Additionally, RSV seems to reduce the clinical feature of RA, with reduced paw swelling throughout the time, and histologically, it seems to promote articular protection.

IL-4 is an anti-inflammatory cytokine and it is well known that its concentration is reduced in periodontal compromised patients [53–55]. This cytokine is an inhibitor of production of pro-inflammatory cytokines TNF-α, IL-1α, IL-1β, IL-6 and IL-8 [56, 57]. IL-4 is also a B cell stimulatory factor and promotes immunoglobulin (Ig) class switching to IgE [58]. Thus, low level of IL-4 results in higher periodontal destruction and higher levels act as protective role in periodontitis. Higher levels of IL-4 promotes the regulation of immune function and reduced macrophage survival in the inflammatory lesion. In the present study, it was observed lower alveolar bone loss when ibuprofen and resveratrol were administered. Additionally, the results showed modulatory effect of RSV in IL-4 levels, which can explain the lower bone loss in the RSV group, as showed in a previous study of our research group [36] where the systemic treatment of resveratrol increased the gingival levels of IL-4 in a rat ligature-induced periodontitis model. Moreover, IL-4 levels were higher at RSV-treated group on the periodontitis-induced gingival tissue when compared to the non-periodontitis-induced tissue, confirming the protective effect of this marker. Ibuprofen did not modulate IL-4 levels, nor the other cytokine levels in our study, suggesting that the pathway by ibuprofen reduces periodontal breakdown may be through another mechanism not investigated in the study. A periodontitis beagle model in which ibuprofen was systemic applied resulted in reduced levels of PGE_2_ and thromboxane in gingival fluid and lowered alveolar bone loss when compared to the control animals [59]. Besides, although the association of ibuprofen and other non-steroidal anti-inflammatory drugs to periodontal non-surgical therapy have been demonstrated as a positive approach [60], the use of this type of drug can result in several adverse effects, as gastrointestinal problems, bleeding (as a result of decreased platelet aggregation), renal and hepatic impairment. In addition, since there is cessation of ingestion of these drugs, there may be a return or rebound effect on the rate of bone loss [61].

The modulation of IL-4 levels by resveratrol it is an important finding regarding the progression of rheumatoid arthritis. It has been shown that IL-4 reduces joint inflammation and bone destruction [62–64]. Besides, IL-4 secreted by dendritic cells suppressed IL-17 production by T cells in the early phase of CIA, reducing the incidence and severity of the disease [62, 65]. In addition, IL-4 was related to the inhibition of IL-1β production and to the increasing production of IL-1 receptor antagonist in rheumatoid synovial explants. Taken together, these results may be related to the reduction of inflammatory signs of RA in the present study represented by the reduction in paw edema at the 28^th^ and to the articular improvement seen in the histological findings in the RSV group.

Our results show no difference regarding the levels of the other cytokines analyzed (IL- β 1, IL-6, IL-17, TNF-α) among the groups and this may be related to the coexistence of both periodontitis and rheumatoid arthritis, which may exacerbate the immune-inflammatory response and the administered substances could not overcome this effect. Our research group showed an upregulation of IL-17 with the association of both diseases in rats [13] and it is well known that Th17 plays an important role in the pathogenesis of collagen-induced arthritis, inducing the production of IL-1, TNF-α, IL-6, IL-8, GM-CSF and PGE2 [63, 66–68], as well as in the periodontitis development leading to osteoclastogenesis [69–71]. In the present study, no significant difference in the levels of IL-17 was showed even with the administered treatments and it may be hypothesized that resveratrol and ibuprofen were not able to modulate the level of this marker due to the elevated levels of IL-17 released by the association of both pathologies.

In line, resveratrol modulated the serum levels of Rheumatoid factor and the local levels of anti-citrullinated protein antibody. Both markers are related to increased disease activity and severity [72–74]. Interestingly, studies have showed that RF influences the size of bone erosions on an ACCPA background in a dose dependent manner [75]. Additionally, systemic bone mineral density in patients with early RA is reduced in relation with ACCPA positivity and high RF levels. An animal study suggested that the elevated local levels of ACCPA observed when RA and EP were associated would contribute to periodontal destruction [13]. In accordance with our results, Whaba et al. [76] observed a significant reduction in RF serum levels of arthritic rats improving AR progression. These findings can help to explain the lower bone loss, as well as, the reduction in paw edema and the articular damage in the RSV group. Although ibuprofen has reduced the local levels of ACCPA to a lower level than the reduction promoted by RSV, RF levels were not modulated by it. Thus, the control of the paw edema in ibuprofen group did not extended to the end of the experiment. At the same way, the articular improvement observed histologically was not compared to that seen in RSV group.

Both IB and RSV reduced the levels of ACCPA in the present study. This finding can be related to the lower periodontal destruction in the related groups once the citrullination process alters the complement system activity, inactivates epidermal growth factor and induces prostaglandin E_2_ (PGE_2_) production [77–80], leading to a possible higher periodontal destruction. Additionally, it was also shown a directly association between alveolar bone loss and ACCPA levels [13]. It can be hypothesized that both substances could act reducing the citrullination process. With the above evidence, it can be suggested that both substances promoted protective role in periodontal tissues regarding ACCPA local influence.

The results of the present study can suggest a potential of host modulation by resveratrol when both conditions coexist. The reduction in IL-4 gingival levels may reduce the periodontal breakdown as well as the progression of rheumatoid arthritis. Besides, resveratrol modulated the serum levels of RF and the local levels of ACCPA, which can result in reduction of activity and severity of RA. Additionally, lower ACCPA gingival levels can also reduce the periodontal destruction. Interestingly, a recent clinical trial administered 1g/day of resveratrol with the conventional treatment of RA for 3 months observing reduction in clinical markers, disease activity and inflammatory markers. The author think that different doses should be used to test the effect of lower doses both in RA alone and in conditions of association of both diseases [81]. The observed modulatory effects of resveratrol in the presence of the diseases can suggest the use of a clinical approach for patients who present both conditions and clinical studies evaluating different doses should be conducted to confirm these findings.

Considering the limits of this study, it can be concluded that resveratrol can reduce periodontal destruction and ACCP local levels in the presence of rheumatoid arthritis. However only resveratrol showed modulatory effect on the levels of IL-4 and rheumatoid factor in arthritic rats and reduce the inflammatory signs of arthritis and articular damage throughout the time.

## Supporting information

S1 File Supporting information. (XLSX)
